# Construction of pH-Sensitive Multifunctional Hydrogel with Synergistic Anti-Inflammatory Effect for Treatment of Diabetic Wounds

**DOI:** 10.3390/pharmaceutics17050644

**Published:** 2025-05-13

**Authors:** Xiaoyan Sun, Yan Li, Haifeng Wang, Yanqiu Meng, Xu Dai, Lina Du, Lei Li

**Affiliations:** College of Chemical Engineering, Shenyang University of Chemical Technology, Shenyang 110142, China

**Keywords:** diabetic wounds, macrophages, hydrogel, self-healing, anti-inflammatory

## Abstract

**Background/Objectives:** A sustainable inflammatory response is a significant obstacle for diabetic wound care. In this study, the pH-sensitive multifunctional hydrogel ODex/BSA-Zn was fabricated via a Schiff base and coordination force for the first time. **Methods:** The hydrogel consisted of oxidized dextran (ODex), bovine serum albumin (BSA), and zinc ions (Zn^2+^) in the absence of an additional crosslinking agent. **Results:** The hydrogel showed excellent mechanical stability, fast self-healing ability, and significant anti-inflammatory effects, as demonstrated by the formation of dynamic covalent bonds between the aldehyde group (-CHO) of ODex and the amino group (-NH_2_) of BSA via the Schiff base reaction, as well as the metal-ion coordination reaction of Zn^2+^ with the imidazole ring of BSA. In a diabetic mouse full-thickness cutaneous defect wound model, the ODex/BSA-Zn hydrogel could effectively inhibit the inflammatory response and increase collagen deposition, thereby accelerating the transition of macrophage M1 to M2 and promoting wound closure. This study offers a promising therapeutic approach for managing long-term diabetic ulcers.

## 1. Introduction

Diabetes mellitus (DM) is an increasingly prevalent chronic metabolic disease characterized by prolonged hyperglycemia, with impaired wound healing representing one of its severe complications [1,2]. Wound healing is an evolutionarily conserved process involving inflammation, clotting, cell proliferation, and extracellular matrix (ECM) restructuring [3,4,5]. In diabetic wounds, a persistent hyperglycemic microenvironment leads to the excessive accumulation of advanced glycation end-products (AGEs) [6] and induces the overexpression of ROS [7], which disrupts the balance in wound healing and sustains chronic inflammation [8,9]. M2 macrophages demonstrate anti-inflammatory properties and facilitate angiogenesis and collagen deposition [10], but the prolonged chronic inflammatory environment causes the transformation failure of M1 macrophages to M2, thereby hindering wound healing [11,12,13]. Therefore, shifting macrophage polarization from M1 to M2 via inflammatory factor manipulation offers a viable therapeutic strategy for diabetic wound healing [14,15].

Bovine serum albumin (BSA) has a similar structure to human albumin, with good mechanical properties, biocompatibility, and degradability [16]. Modern studies have demonstrated that BSA can improve wound healing and enhance tissue repair via antioxidant activity and inflammatory regulation [17,18]. In addition to direct administration in wound regions, the abundant carboxyl and amino groups in BSA can interact with metal ions to form a protein network via salt bridge formation. Zinc is a crucial trace element within the human body [19]. Its indispensable function is vital for sustaining typical bodily functions and health. In the wound microenvironment, zinc ions (Zn^2^⁺) exert a pivotal role by suppressing inflammatory factors, scavenging reactive oxygen species (ROS), enhancing antioxidant enzyme activity, inhibiting pro-oxidative pathways, and regulating immune cell functions. By downregulating the expression of antioxidant genes (SOD1, SOD2), it diminishes the cellular requirements for oxidative stress defense, indirectly mitigating inflammation. Furthermore, Zn^2+^ suppresses NF-κB activation, which decreases the secretion of pro-inflammatory cytokines (such as TNF-α and IL-1β), thereby attenuating inflammatory responses [20,21]. Compared with conventional therapy strategies, zinc ion treatments display many advantages such as low cost, simplicity, and favorable stability [22]. Therefore, we hypothesized that Zn^2+^ can interact with BSA to form a protein network and enhance drug delivery capability. However, simple BSA-Zn^2+^ networks struggle to achieve favorable therapeutic outcomes due to their unsatisfactory retention time and fast elimination [23]. Therefore, a novel strategy urgently needs to be developed.

Hydrogels are hydrophilic polymeric materials characterized by three-dimensional network structures, exhibiting high water content, flexibility, and excellent biocompatibility [24,25]. Owing to their superior tissue exudate absorption and gas permeability for wound moisture retention, hydrogels have emerged as ideal materials for wound dressings and tissue regeneration [26,27]. However, traditional hydrogels lack mechanical strength and are prone to breakage, hindering controlled drug delivery [14]. Therefore, multifunctional hydrogels are ideal for targeted drug delivery due to their site-specific release capabilities [28,29]. Recently, many stimulus-responsive hydrogels have been developed, such as those responding to pH [30], enzymes [31], glucose [32], ROS [33], and temperature, which can effectively facilitate wound healing [34]. Among different types of stimulus-responsive hydrogels, Schiff-base self-healing hydrogels formed with aldehyde groups and amino groups via a nucleophilic addition reaction [35] have demonstrated remarkable mechanical properties and autonomous repair capabilities, representing a promising strategy for diabetic wound management [36,37,38]. Schiff-base hydrogels are stable at a physiological pH and become hydrolyzed at an acidic pH to release therapeutic drugs for wound healing. Meanwhile, the reversibility of dynamic Schiff base bonds can also allow hydrogels to be self-healing [39] and shear thinning, which can improve hydrogel adaption to the wound region [40]. Dextran is a biocompatible polysaccharide that has been widely used as a plasma volume expander and was approved by the FDA [41,42]. The hydroxyl groups of dextran can be oxidized to aldehyde groups, which can interact with amino group-rich substitutes to form dynamic Schiff base bonds. Meanwhile, abundant aldehyde groups can also increase adhesion with soft tissue. Therefore, oxidized dextran may interact with the amino groups of BSA and form a dynamic crosslinking network.

**Scheme 1:**
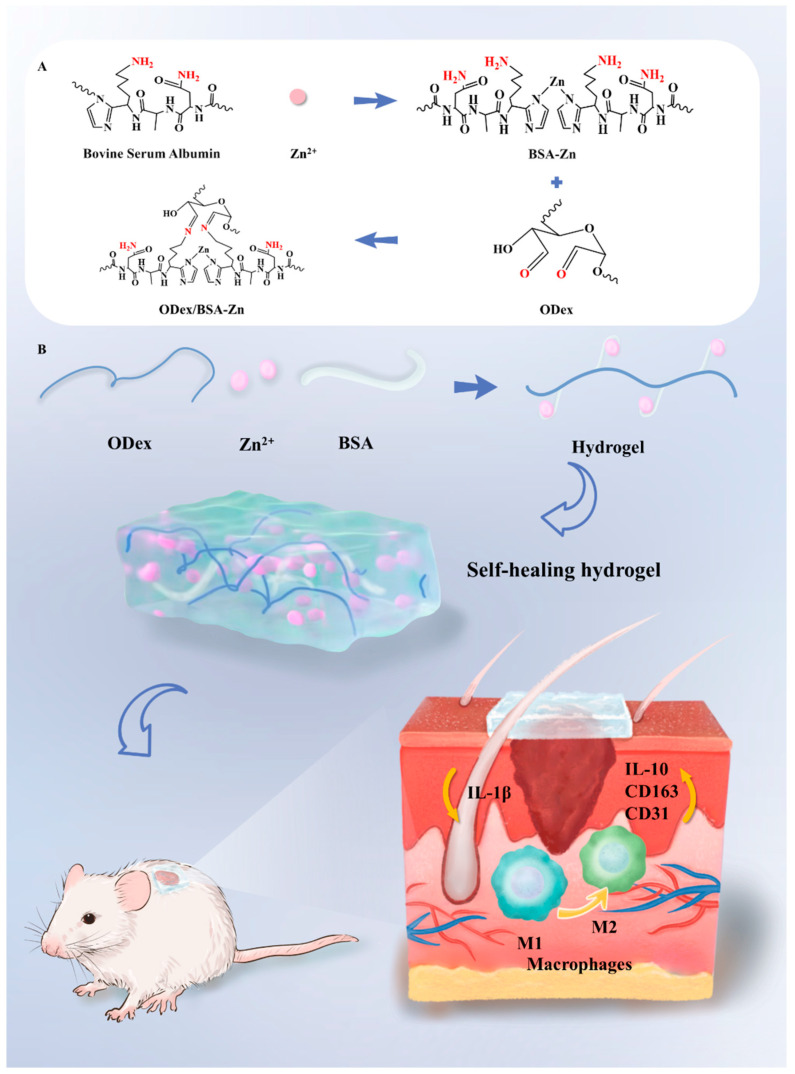
Diagram Illustrating of the ODex/BSA-Zn Hydrogel Platform for Diabetic Wound Healing. (**A**) Formation mechanism of ODex/BSA-Zn hydrogel. (**B**) Mechanism of ODex/BSA-Zn hydrogel in promoting wound healing in diabetic mouse.

In this paper, a double-network self-healing hydrogel based on oxidized dextran/bovine serum albumin/Zn^2+^ was fabricated (ODex/BSA-Zn). Zn^2+^ was first reacted with BSA to form a BSA/Zn^2+^ protein network via salt bridge formation. Subsequently, aldehyde groups provided by oxidized dextran interacted with the amino groups of bovine serum albumin to form Schiff base bonds, thereby endowing the hydrogel with self-healing capabilities [38] (Scheme 1A). The aldehyde groups provided by oxidized dextran and the amino groups of bovine serum albumin form dynamic chemical bonds through Schiff base reactions. In the early stage of diabetic wound healing, the acidic microenvironment [6] allows the self-healing hydrogel to release Zn^2+^ to decrease the expression of inflammatory factors, accelerating the conversion of M1 macrophages to M2 macrophages [43] (Scheme 1B). The hydroxyl groups in the dextran are protonated in the weakly acidic environment to maintain the chemical balance in the wound microenvironment. Compared with traditional multifunctional hydrogels, this hydrogel has the following advantages: (1) dual-network hydrogel was prepared via simple physical mixing without additional catalysts or crosslinking agents; (2) the materials used to prepare the hydrogel all have good biocompatibility and biodegradability; (3) the self-healing characteristic formed by dynamic bonds can make the hydrogels completely cover the wound, and it can still restore them to their original state even under external force. Overall, the rationally designed self-healing hydrogel represents a facile and effective strategy for diabetic wound therapy.

## 2. Materials and Methods

### 2.1. Materials

Dextran (Dex, Mw = 70,000 Da) and sodium periodate (NaIO_4_, 99.5%) were obtained from Shanghai Macklin Biochemical Co., Ltd., Shanghai, China. Bovine serum albumin (BSA, Mw: 66.430 KDa) was purchased from Beijing Solarbio Technology Co., Ltd., Beijing, China. Dialysis membranes with a molecular weight cut-off (MWCO) of 3500 Da were supplied by Beijing Biotopped Science and Technology Co., Ltd., Beijing, China. Ethylene glycol (CH_2_OH)_2_, zinc chloride (ZnCl_2_), potassium dihydrogen phosphate (KH_2_PO_4_), disodium hydrogen phosphate (Na_2_HPO_4_), sodium chloride (NaCl), and potassium chloride (KCL) were procured from Tianjin Hengxing Chemical Preparation Co., Ltd., Tianjin, China. All the other chemical solvents and reagents were of analytical grade and used without further purification.

### 2.2. Preparation of Oxidized Dextran (ODex)

The preparation of ODex was carried out according to the literature [44,45,46]. Briefly, 13.2 g of sodium periodate was gradually added to 100 mL of a 10% (*w*/*v*) dextran solution in water under continuous stirring. The reaction mixture was stirred for 5 h at room temperature in the dark. Subsequently, 2 mL ethylene glycol was added to quench the reaction. The resulting solution was transferred into a dialysis bag with a molecular weight cut-off (MWCO) of 3500 Da and dialyzed with distilled water at 4 °C for 3 days After dialysis, the solution was lyophilized to yield ODex. The structure of the ODex was characterized by FT-IR (Nicolet iS50 FTIR Thermo Fisher Scientific, Waltham, MA, USA) and UV-Vis (Agilent Cary 5000, Agilent Technologies Inc, Santa Clara, CA, USA).

### 2.3. Preparation and Characterization of the Hydrogels

A 10% (*w*/*v*) ODex solution was prepared by dissolving 0.2 g of ODex in 2 mL of distilled water. Separately, 0.4 g of BSA was dissolved in 2 mL of distilled water, and 2 mL of a 0.15% (*w*/*v*) ZnCl₂ solution to prepare 20% (*w*/*v*) BSA and 20% (*w*/*v*) BSA-Zn solutions. Then, 2 mL of the ODex solution was combined with each of the BSA and BSA-Zn solutions, respectively. The mixtures were gently stirred and then left undisturbed at room temperature to allow hydrogel formation, during which the gelation time was noted. The resulting hydrogels were designated as ODex/BSA and ODex/BSA-Zn. Their structural characteristics were analyzed using FT-IR spectroscopy and scanning electron microscopy (SEM).

### 2.4. Swelling and Degradationcharacteristics of the Hydrogels

Swelling test: Two freeze-dried hydrogel samples were immersed in distilled water for two hours after lyophilization. The initial dry weight (*m*_0_) of each sample was measured before immersion. At predetermined time intervals, the hydrated weight (*m_i_*) was recorded. The swelling ratio (SR) of the hydrogels was calculated using using the following equation [38]:

$$\mathrm{SR}\;(\%)=\frac{m_{i}-m_{0}}{m_{0}}\;\times \;100$$

Degradation test: To evaluate in vitro degradation under mildly acidic conditions (pH = 6), hydrogels with identical initial weights (*m*_0_) were submerged in 10 mL of phosphate-buffered saline (PBS) and incubated at 37 °C. At predetermined time intervals, samples were removed, and their remaining mass (*mₜ*) was measured. The degradation ratio (DR) of the hydrogels was calculated using the following equation [47]:

$$\mathrm{DR}\;(\%)=\frac{m_{t}}{m_{0}}\;\times \;100$$

### 2.5. Release Profile of Zn^2+^

The release behavior of Zn^2^⁺ from the hydrogel was evaluated by cylindrical ODex/BSA-Zn hydrogel samples (approximately 2 cm in diameter and 2 mm thick). Each sample was incubated at 37 °C with 2 mL of PBS at pH = 6 or pH = 7.4 added to the top of the hydrogel. At predetermined time intervals, the release medium was collected and replaced with an equal volume of fresh PBS. The amount of Zn^2^⁺ released from the ODex/BSA-Zn hydrogel was quantified through a colorimetric reaction with Eriochrome Black T. The absorbance of the sample solution was measured at 538 nm using a UV-Vis spectrophotometer.

### 2.6. Rheological Characterization of the Hydrogels

The rheological characterization of the ODex/BSA-Zn hydrogels was assessed using a rheometer (TA Instruments AR2000ex, TA Instruments, New Castle, DE, USA). Time-dependent measurements were performed at a constant frequency of 1 rad/s and a fixed strain of 1%. Additionally, frequency-sweep tests were conducted over a range of 0.1 to 100 rad/s, at 1% strain. To evaluate the self-healing capability of the hydrogels, a step-strain cycling test was implemented, alternating between low strain (γ = 1%) and high strain (γ = 1000%) for three consecutive cycles.

### 2.7. Cytotoxicity Evaluation of the Hydrogels

The cytotoxicity of the hydrogels was assessed using the MTT assay on L929 mouse fibroblast cells. Briefly, cells were then placed in the 96-well plate at a cell density of 1.5 × 10^3^ cells/100 μL per well and incubated at 37 °C for 24 h. After incubation, the culture medium was replaced with conditioned medium. Cells cultured with fresh medium without any hydrogel treatment served as the control group. The conditioned medium was prepared by incubating ODex/BSA-Zn and ODex/BSA hydrogels in DMEM at a mass ratio of 1∶5 for 1, 3, and 5 days, respectively. Cell viability was then determined using the MTT assay.

### 2.8. In Vitro Anti-Inflammatory Activity Evaluation

The anti-inflammatory activity of the hydrogel was evaluated by measuring the levels of the pro-inflammatory factor IL-1β and anti-inflammatory factor IL-10 secreted by RAW 264.7 macrophages upon simulation with lipopolysaccharide (LPS) [48]. RAW 264.7 macrophages were added to 6-well plates at a density of 1 × 10^6^ cells/mL, and incubated with DMEM containing LPS (10 μg/mL) for 2 h. After LPS simulation, the cells were further incubated for 24 h with 2 mL of diluted ODex/BSA hydrogel, ODex/BSA-Zn hydrogel, and DMEM, respectively. The supernatants were then collected and analyzed using ELISA to quantify IL-1β and IL-10 levels.

### 2.9. In Vivo Evaluation of Diabetic Wound Healing

The therapeutic efficacy of hydrogels in diabetic wound healing was evaluated using ICR mice (20–25 g, 4–6 weeks old), and after a 12-hour fast, Type 1 diabetes was induced by intraperitoneal injection of streptozotocin (STZ) for 7 consecutive days. One week following the final STZ injection, mice with a blood glucose level > 16.7 mmol/L were considered as successfully developed diabetic mice [30]. A full-thickness, round wound about 8 mm in diameter was then created on the dorsal surface of each mouse. Subsequently, the wounds were then defined as Untreated (negative control) or treated with Tegaderm (positive control), ODex/BSA, or ODex/BSA-Zn hydrogel in urea cream, respectively. Treatments were administered daily, with the hydrogel dressings changed accordingly. Wound sites were photographed on Days 0, 3, 7, and 14, and wound measurements were recorded using Image J 1.54k software. The wound healing rate was calculated using the formula: 

Wound Healing Rate (%) = $\frac{A_{0}-A_{t}}{A_{0}}$ × 100,

where *A*_0_ is the area of the wound on Day 0 and *A_t_* is the area of the wound at the specified time.

### 2.10. Histology and Immunofluorescence Staining Analysis

Tissue regeneration was characterized through histopathological examination of wound samples. At designated time points, wound tissues were collected, fixed in 4% paraformaldehyde, processed into paraffin blocks, and sectioned into 5 μm slices. These sections were stained with hematoxylin–eosin (H&E) and Masson trichrome, and examined under an optical microscope (Olympus BX43, Olympus, Tokyo, Japan) to evaluate tissue morphology and collagen deposition. After 7 days of treatment, blood samples were collected from the tail vein and centrifuged to isolate serum for quantification of IL-1β levels using an ELISA kit (MeilunBio, Dalian, China). To assess the in vivo biocompatibility of the hydrogels, the main organs (heart, liver, spleen, lungs, and kidney) were excised 14 days post-treatment and subjected to H&E staining for histological analysis. Moreover, immunofluorescent staining was performed for IL-1β, CD163, and CD31 following standard protocols. Stained sections were observed using an inverted fluorescence microscope (Olympus BX43, Olympus, Tokyo, Japan), and the fluorescence intensity was quantitatively analyzed with ImageJ 1.54k software.

### 2.11. Statistical Analysis

All results are expressed as mean ± standard deviation (SD), with a minimum of three independent replicates (*n* ≥ 3). Significant differences were assessed using multiple comparisons ANOVA (GraphPad Prism 8), with *p* < 0.05 defined as statistically significant (NS defined as not statistically significant, * *p* < 0.05, ** *p* < 0.01, *** *p* < 0.001).

## 3. Results and Discussion

### 3.1. Characterization of Oxidized Dextran

**Figure 1:**
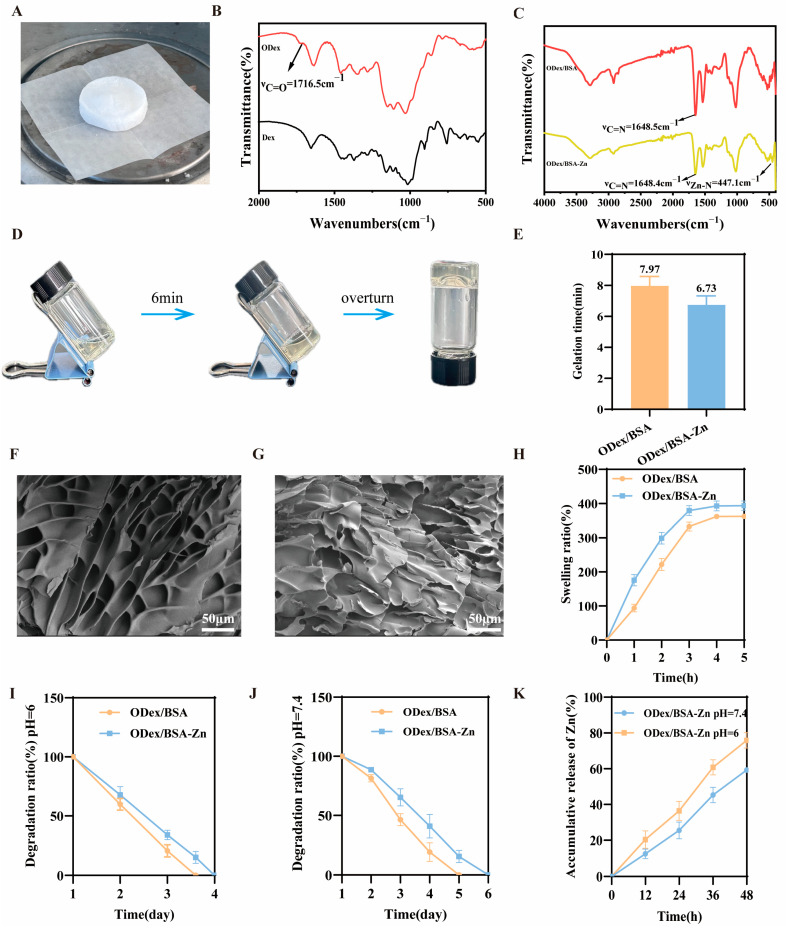
Oxidized dextran, hydrogel structure, and hydrogel performance test results. (**A**) Image showing the visual appearance of oxidized dextran. (**B**) FT-IR spectrum of ODex. (**C**) FT-IR spectrum of hydrogel. (**D**) Photograph of the hydrogel. (**E**) Gelation time of hydrogels (*n = 3*). (**F**) Representative SEM images of ODex/BSA-Zn hydrogels (scale bars = 50μm). (**G**) Representative SEM images of ODex/BSA hydrogels (scale bars = 50 μm). (**H**) Swelling ratio of hydrogels (*n = 3*). (**I**) Degradation ratio of hydrogels in pH = 6 at 37 °C (*n = 3*). (**J**) Degradation ratio of hydrogels in pH = 7.4 at 37 °C (*n = 3*) (**K**) Accumulative release of Zn^2+^ from ODex/BSA-Zn hydrogels in PBS (pH = 6 and 7.4) *(n = 3*).

ODex was obtained by sodium periodate oxidation, and subsequently freeze-dried, resulting in a sponge-like morphology as shown in Figure 1A. The chemical structure of the ODex was confirmed by FT-IR (Figure 1B), exhibiting a characteristic absorption peak at 1716.5 cm^−1^, corresponding to the C=O stretching vibration of the aldehyde group. These results confirmed the successful synthesis of ODex.

### 3.2. Fabrication and Characterization of the ODex/BSA-Zn Hydrogel

The dynamic Schiff base reaction was formed by the aldehyde group (-CHO) of the ODex and the amino group (-NH_2_) of the BSA, while Zn^2+^ ions coordinated with the imidazole rings of the BSA to improve the strength of the hydrogel’s mechanical strength. This dual interaction resulted in a dual-network self-healing hydrogel. As shown in Figure 1C, the FT-IR spectra displayed a characteristic peak at 1648 cm^−1^, indicating the formation of a C=N bond through a Schiff base reaction in both hydrogel samples. Furthermore, the ODex/BSA-Zn hydrogel exhibited a distinctive low-frequency absorption peak at 447 cm^−1^, indicative of metal coordination [19,49]. All the above data confirmed the formation of a Schiff base and metal coordination bonds in the ODex/BSA-Zn hydrogel. Figure 1D illustrates the morphological transition during gelation, comparing the precursor solution to the formed hydrogel network. Upon the addition of Zn^2+^, the gelation time significantly reduced to 1.24 min. This acceleration is attributed to the rapid coordination between Zn^2+^ ions and BSA, enabling the swift formation of a stable cross-linked network (Figure 1E). The SEM results revealed in Figure 1F,G that, compared to the ODex/BSA hydrogel, the ODex/BSA-Zn hydrogel exhibited a more porous structure. This enhanced porosity allows for better permeability, promoting fibroblast migration and the transport of essential nutrients such as oxygen, factors that are crucial for accelerating diabetic wound healing.

Swelling capacity was assessed by measuring expansion rates over 5 h (Figure 1H). Both hydrogel samples reached swelling equilibrium within 3 h. Notably, the ODex/BSA-Zn hydrogel exhibited the highest swelling ratio, reaching up to 393.67%. This superior performance may be due to its porous three-dimensional structure, which enhances liquid absorption. Such a high absorption capacity makes this hydrogel particularly effective at treating wound exudates, keeping the wound bed clean and promoting better healing conditions.

The microenvironment of diabetic wounds is typically acidic due to the byproducts of bacterial respiration and fermentation. To assess the performance of the two hydrogel samples under such conditions, the degradation behavior and Zn^2+^ release rate from the ODex/BSA-Zn hydrogel were evaluated in PBS (pH = 6 and 7.4). The ODex/BSA-Zn hydrogel demonstrated a slower degradation ratio compared to the ODex/BSA hydrogel, probably due to its significantly higher internal cross-linking density structure (Figure 1I,J). Meanwhile, the Zn^2+^ release rate in Figure 1K illustrates a significantly faster release under acidic conditions, attributed to the protonation of imidazole nitrogen groups in BSA, which promotes Zn^2+^ release. As a result, in acidic conditions, the ODex/BSA-Zn hydrogel combines slower degradation and accelerated Zn^2+^ release, which are crucial for wound healing.

### 3.3. Rheological Characterization of ODex/BSA-Zn Hydrogel

**Figure 2:**
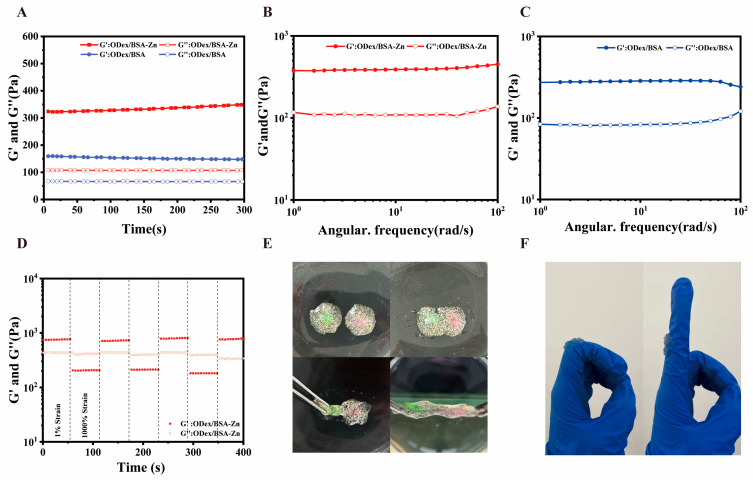
Rheological properties of hydrogel. (**A**) Hydrogel time rheogram. (**B**) ODex/BSA-Zn hydrogel frequency rheogram. (**C**) ODex/BSA hydrogel frequency rheogram. (**D**) Rheological behavior of ODex/BSA-Zn hydrogel over three cycles between 1 and 1000% strain. (**E**) Picture of hydrogel self-healing ability. (**F**) Picture of hydrogel adhesion.

Essential for hydrogels to adequately protect wounds are optimal mechanical and regeneration characteristics [50]. In this study, the rheological behavior of ODex/BSA and ODex/BSA-Zn hydrogels were evaluated by monitoring the changes in storage modulus (G′) and loss modulus (G″) over time under constant frequency (1 Hz) and strain (1%) conditions. As depicted in Figure 2A, the storage modulus (G′) was consistently exceeded by the loss modulus (G″), indicating the favorable structural stability of both the ODex/BSA-Zn and ODex/BSA hydrogels. Owing to its higher cross-linking density, the ODex/BSA-Zn hydrogel exhibited the higher G′ value, reaching up to 348 Pa. Furthermore, within the frequency range of 0 to 100 rad/s, G′ remained consistently higher than G″ without any crossover, further confirming the excellent stability of both hydrogels (Figure 2B,C).

The formation of dynamic Schiff bases endows these hydrogels with the capability to rapidly restore their structure following deformation caused by external forces. This self-healing behavior was assessed using sequential step-strain sweeps. As demonstrated by the strain scan results of the ODex/BSA-Zn hydrogel (Figure 2D), a high strain of 1000%, caused G′ to drop below G″, indicating internal structure disruption. However, upon reducing the strain back to 1%, both G′ and G″ values returned to their initial state. After three cyclic tests, the modulus values showed negligible values, highlighting the excellent self-healing properties of the ODex/BSA-Zn hydrogel. The reversible crossover of G′ and G” confirmed the material’s shear-thinning properties and dynamic self-healing capacity, providing a solid theoretical and practical foundation for potential clinical applications in treating complex wound regions.

To further investigating the self-healing capabilities of hydrogel, two hydrogels of different colors were placed in contact. A fewer minutes later, the hydrogels were able to repair themselves, with no visible fractures at the junction (Figure 2E). In Figure 2F, the ODex/BSA-Zn hydrogel also exhibited strong adhesion to the skin. The reversible dynamic Schiff base bonds and this robust adhesion are key features of the ODex/BSA-Zn hydrogel, making it a highly promising candidate for experimental studies in diabetic wound healing.

### 3.4. In Vitro Cytotoxicity and Anti-Inflammatory Activity of ODex/BSA-Zn Hydrogel

**Figure 3:**
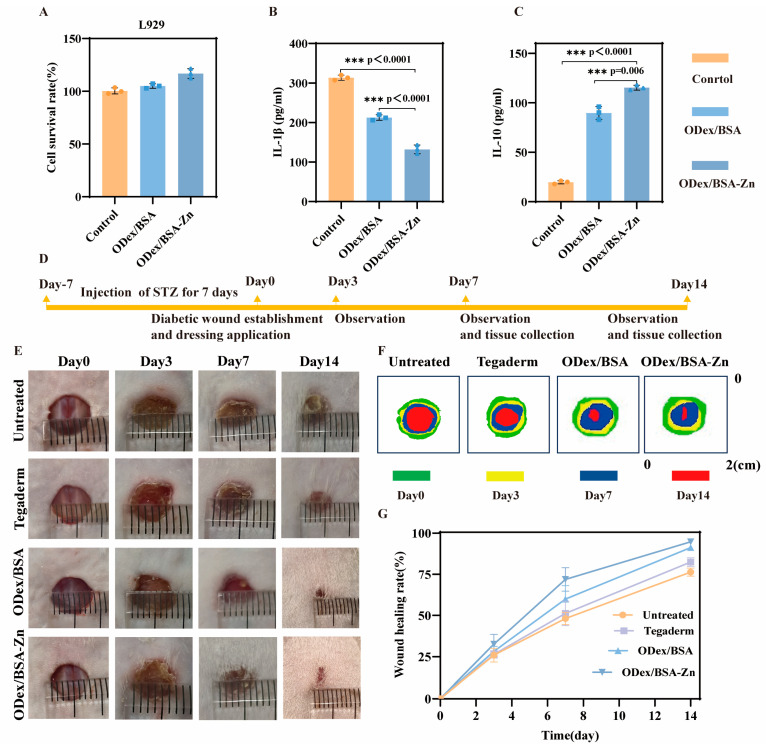
In vitro cytotoxicity and anti-inflammatory activity of ODex/BSA-Zn hydrogel and in vivo wound healing of ODex/BSA-Zn hydrogel. (**A**) Mouse fibroblast survival. (**B**) Pro-inflammatory factor IL-1β content in mouse macrophage supernatants. (**C**) Anti-inflammatory factor IL-10 content in mouse macrophage supernatants. (**D**) Illustration of wound therapy for diabetic mice. (**E**) Wound images under various treatments at Days 0, 3, 7, and 14. (**F**) Traces of wound healing. (**G**) Quantitative analysis of wound healing rate based on wound area. Data are expressed as means ± SD. *** *p* < 0.001.

The cytocompatibility of the hydrogel with L929 cells was evaluated using the MTT assay, as illustrated in Figure 3A. Following a 72-h incubation period, the cells exhibited excellent viability with no signs of toxicity, confirming the hydrogel’s biocompatibility. Notably, the addition of Zn^2^⁺ did not affect its biocompatibility.

To investigate the anti-inflammatory properties of the ODex/BSA-Zn hydrogel, we examined inflammatory factors in LPS-activated RAW264.7 mouse macrophages after adding hydrogel samples. The supernatant was collected and IL-10 and IL-1β secretion levels were both measured using ELISA. As depicted in Figure 3B, the control group exhibited higher levels of IL-1β in the supernatant, while the ODex/BSA-Zn hydrogel group displayed the lowest levels compared to both the ODex/BSA hydrogel group and the control group. This result suggests that Zn^2+^ effectively suppresses the expression of the pro-inflammatory factor IL-1β. Conversely, as illustrated in Figure 3C, the ODex/BSA-Zn hydrogel group demonstrated the highest levels of IL-10 secretion, indicating that using hydrogel enhanced the production of the anti-inflammatory factor IL-10. Collectively, the statistical analysis of inflammatory factors confirms that the ODex/BSA-Zn hydrogel effectively decreases pro-inflammatory cytokines while elevating anti-inflammatory mediators, thereby establishing a robust basis for subsequent in vivo wound healing applications.

### 3.5. In Vivo Wound Healing of ODex/BSA-Zn Hydrogel

In this study, assessment of the wound healing efficacy of ODex/BSA-Zn hydrogel was evaluated using a diabetic mice model with full-thickness skin defects. Hydrogel samples were applied continuously over a 14-day period, and the defect locations were photographed on Day 3, 7, and 14 of application, as detailed in Figure 3D. Macroscopically, wounds treated with ODex/BSA-Zn and ODex/BSA hydrogels healed noticeably quicker than those covered with Tegaderm, which might be attributed to the hydrogel’s ability to maintain localized moisture and actively promote the wound healing process [37,40] (Figure 3E). Figure 3F schematically presents the change in wound area versus time, clearly reflecting the superior healing effects of ODex/BSA-Zn [20]. Taking the above, these findings demonstrate that the hydrogel’s properties and the material’s anti-inflammatory effects were key factors in speeding up the healing process [21].

Based on the wound healing rate data shown in Figure 3G, after 7 days of treatment, the wound healing rate of the Untreated group and Tegaderm group were 48.11% and 51.25%, and that of the ODex/BSA hydrogel group and ODex/BSA-Zn hydrogel group were 59.96% and 71.87%. These results demonstrate that both hydrogel formulations significantly outperformed the control groups. After 14 days, the wound healing rate of the Untreated, Tegaderm, ODex/BSA, and ODex/BSA-Zn group was 76.38%, 82.46%, 91.21%, and 94.80%, respectively. This was because the self-healing properties of the hydrogel could better cover the wound, providing protection to the injured area [51]. Additionally, the synergistic anti-inflammatory effects of Zn^2+^ and BSA further contributed to the accelerated tissue repair. Therefore, these results demonstrate that the ODex/BSA-Zn hydrogel was able to effectively promote wound healing via a synergistic anti-inflammatory response in the diabetic wound model.

### 3.6. In Vivo Histological Assessment of Wound Healing

**Figure 4:**
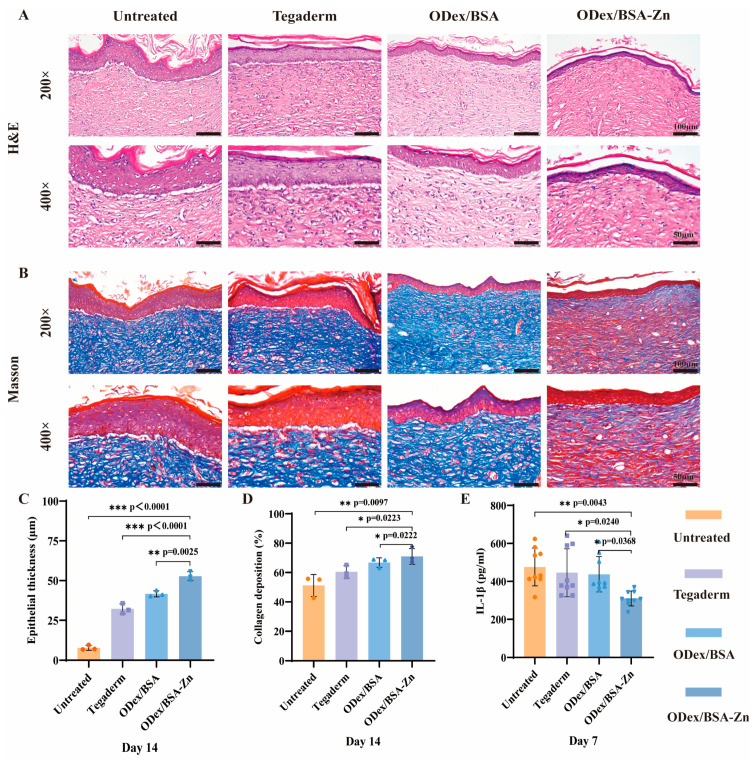
H&E and Masson staining results of diabetic wound models in mice. (**A**) H&E-stained wound sections from each group at Day 14 post-injury, scale bars = 100 μm and 50 μm (*n = 3*). (**B**) Masson-stained wound sections from each group at Day 14 post-injury, scale bars = 100 μm and 50 μm (*n = 3*). (**C**) Quantitative analysis of H&E-stained epithelial thickness on Day 14. (**D**) Quantitative analysis of Masson-stained collagen deposition at Day 14. (**E**) IL-1β cytokines were measured on Day 7. Data are expressed as means ± SD. * *p* < 0.05, ** *p* < 0.01, *** *p* < 0.001.

To evaluate the healing effects of ODex/BSA-Zn hydrogel on diabetic wounds, Figure 4A,B display H&E and Masson’s staining results. As shown in Figure 4C, after 14 days of treatment, the epithelial thickness in the ODex/BSA-Zn hydrogel, the ODex/BSA hydrogel, Tegaderm, and Untreated group were 52.80 ± 2.82 μm, 41.74 ± 1.91 μm, 32.20 ± 3.19 μm, and 7.81 ± 1.52 μm, respectively. In the hydrogel groups, the epithelium appeared to be decreased and to became more compact, indicating enhanced diabetic wound healing. The ODex/BSA-Zn hydrogel group demonstrated densely packed collagen fibers with minimal vacuolated areas and a well-organized structure. Conversely, the Untreated group displayed sparse collagen fibers and abundant vacuolated regions, confirming the superior wound-healing efficacy of the hydrogel treatment. Since collagen plays an essential role in tissue repair, its deposition was evaluated in the reconstructed tissues using Masson staining. As shown in Figure 4D, the ODex/BSA-Zn hydrogel group demonstrated a tight, uniform fiber arrangement with a collagen deposition rate of 70.94 ± 5.35%, significantly higher than the 51.18 ± 7.45% observed in the Control group. The results demonstrate that ODex/BSA-Zn hydrogel significantly accelerates wound healing in diabetic models by enhancing re-epithelialization and collagen deposition, leading to improved therapeutic efficacy.

To assess the inflammatory response in the diabetic wound mouse model, serum cytokine levels were measured. The concentration of the pro-inflammatory cytokine IL-1β was quantified using an ELISA kit, and the result is presented in Figure 4E. According to the result, it can be seen that the content of IL-1β in the hydrogel group was lower than in Tegaderm group, and at the same time, it was lower in the ODex/BSA-Zn hydrogel group than in the ODex/BSA hydrogel group. These results indicate that the hydrogel containing Zn^2+^ inhibits pro-inflammatory factors and exerts anti-inflammatory effects.

### 3.7. Immunofluorescence Staining Analysis

Immunohistochemical staining of wound tissue was conducted to evaluate the expression of IL-1β, CD163, and CD31 to assess inflammatory activity and blood vessel formation within the wound microenvironment. These cytokine expression patterns were examined to better understand the molecular mechanism by which the ODex/BSA-Zn hydrogel promotes faster wound healing.

**Figure 5:**
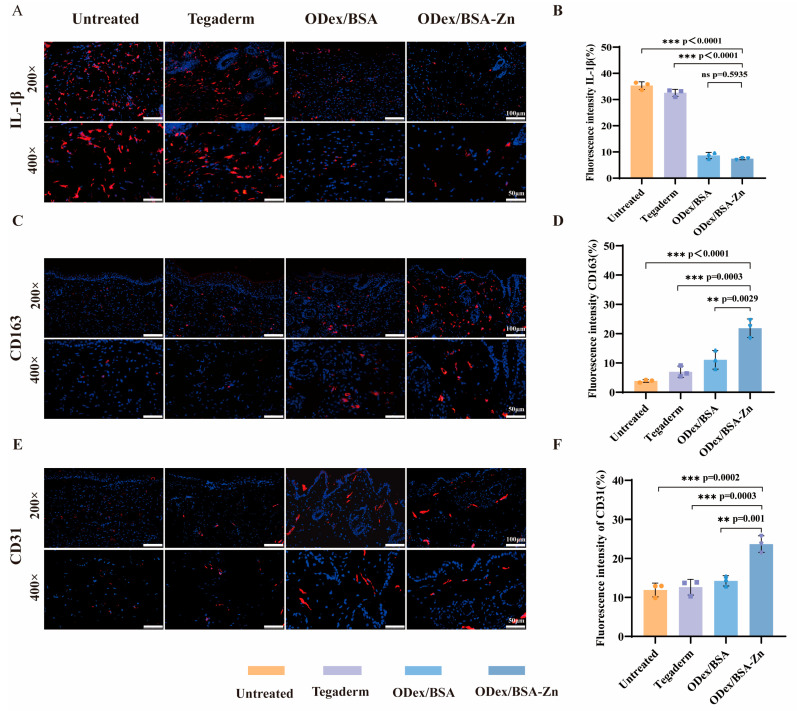
Immunofluorescence Staining Results (**A**) Representative IL-1β immunofluorescence (Day 7 post-injury; *n = 3*). (**B**) IL-1β immunofluorescence staining intensity. (**C**) Representative CD163 immunofluorescence (Day 7 post-injury; *n = 3*). (**D**) CD163 immunofluorescence staining intensity. (**E**) Representative CD31 immunofluorescence (Day14 post-injury; *n = 3*). (**F**) CD31 immunofluorescence staining intensity. Data are expressed as means ± SD. ns as not statistically significant, ** *p* < 0.01, *** *p* < 0.001.

As depicted in Figure 5A, red fluorescence represents inflammatory cytokines. The Untreated and Tegaderm groups exhibited intensively red fluorescence, indicating elevated inflammatory cytokine levels in diabetic wounds. Compared to the Untreated group (35.32 ± 1.47), the ODex/BSA-Zn hydrogel group showed a significant reduction in IL-1β immunofluorescence intensity, quantified at 7.40 ± 0.38 (Figure 5B). 

This result demonstrates that the acidic microenvironment of diabetic wounds facilitates the release of Zn^2+^ from the hydrogel, thereby inhibiting pro-inflammatory cytokines and up-regulating anti-inflammatory effects. These findings are consistent with the previous ELISA kit results. The H&E staining after 14 days of treatment revealed fewer inflammatory cells in the ODex/BSA-Zn hydrogel group compared to the Untreated group, confirming the anti-inflammatory role of Zn^2+^ in reducing inflammatory factors during the wound healing process.

CD163, a hallmark for M2 macrophages, was utilized to assess the transition from M1 to M2 macrophages [52]. In Figure 5C, the ODex/BSA-Zn hydrogel group displayed markedly stronger fluorescence intensity compared to the Untreated and Tegaderm groups. As shown by the quantitative analysis in Figure 5D, the fluorescence intensity of the ODex/BSA-Zn hydrogel group (21.87 ± 3.16) was higher than that of the Untreated group (3.84 ± 0.46), Tegaderm group (6.99 ± 1.92), and ODex/BSA hydrogel group (11.06 ± 3.19). These results suggest that the application of the ODex/BSA-Zn hydrogel effectively promotes the polarization of M1 macrophages toward the M2 phenotype in diabetic wound environments.

Blood vessels play a crucial role in transporting oxygen and nutrients to the wound site to support tissue remodeling. To investigate the pro-angiogenesis effect of the treatment, CD31 endothelial cell labeling via immunofluorescence was performed 14 days post-treatment. As shown in Figure 5E, the hydrogel groups exhibited strong red fluorescence, while only faint signals were observed in the Untreated and Tegaderm groups. In Figure 5F, compared to the Untreated group (12.25 ± 1.53) and Tegaderm group (12.63 ± 2.00), the fluorescence intensity of the ODex/BSA-Zn hydrogel group and ODex/BSA hydrogel group was 23.00 ± 1.98 and 15.59 ± 1.16, respectively, indicating that the ODex/BSA-Zn hydrogel group generated more blood vessels. 

In summary, the immunofluorescence results support the mechanism by which the ODex/BSA-Zn hydrogel promotes wound healing through the suppression of IL-1β cytokines, the induction of M1-to-M2 macrophage polarization, and the stimulation of angiogenesis.

### 3.8. In Vivo Assessment of Systemic Safety

**Figure 6:**
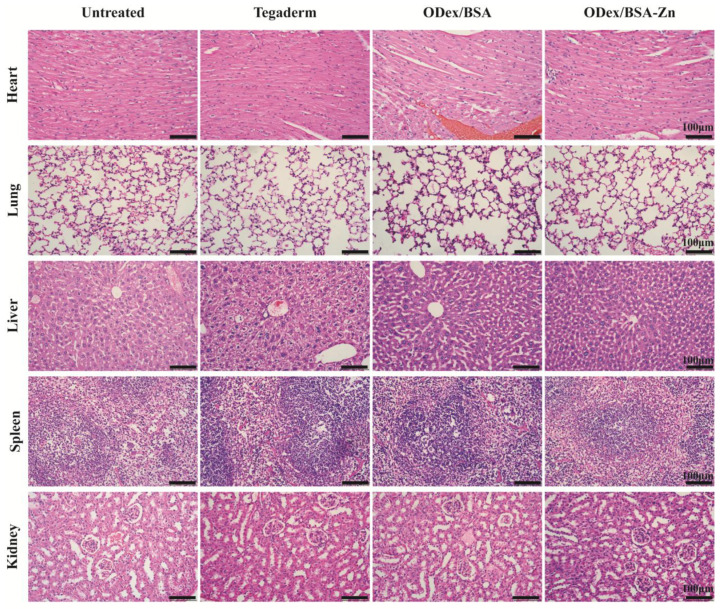
In Vivo Safety Evaluation of ODex/BSA-Zn Hydrogel.

The in vivo safety of the ODex/BSA-Zn hydrogel was also evaluated in this study. Behavioral and histological analyses in major organs were conducted in diabetic mice over a 14-day treatment period. No significant behavioral abnormalities were observed throughout the administration period. At the end of the 14th day, the major organs of the mice were stained using H&E staining (Figure 6). The histological results showed that ODex/BSA-Zn hydrogel administration within the therapeutic did not cause any damage to the mice, conclusively verifying the complete biosafety profile of this therapeutic strategy.

## 4. Conclusions

In summary, a pH- sensitive multifunctional hydrogel was developed for the treatment of chronic diabetic wounds. The ODex/BSA-Zn hydrogel is prepared through a straightforward process that does not require additional cross-linking agents. Its dual network structure is formed via a metal coordination bond (formed by Zn^2+^ and imidazole ring of BSA) and dynamic Schiff base bond (formed by ODex and BSA), endowing the hydrogel with self-healing properties. The hydrogel serves as an efficient delivery platform for Zn^2+^ and BSA at the wound site with superior mechanical strength, self-repair capabilities, and anti-inflammatory efficacy. The ODex/BSA-Zn hydrogel displayed a favorable therapeutic efficacy on the diabetic wound by suppressing inflammatory responses and facilitating collagen deposition and vascular regeneration. Considering these properties and benefits, the ODex/BSA-Zn hydrogel presents a promising strategy for the treatment of diabetic wounds.

## Data Availability

The datasets generated during this study are available from the corresponding author on reasonable request.

## Abbreviations

The following abbreviations are used in this manuscript:

ODex	Oxidized dextran
BSA	Bovine serum albumin
LPS	Lipopolysaccharide
STZ	Streptozotocin
